# Enhanced chlorobenzene removal by internal magnetic field through initial cell adhesion and biofilm formation

**DOI:** 10.1007/s00253-024-13001-z

**Published:** 2024-01-22

**Authors:** Dong-zhi Chen, Jinfeng Qiu, Haimin Sun, Yanting Liu, Jiexu Ye, Jian-Meng Chen, Lichao Lu

**Affiliations:** 1 College of Environment, Zhejiang University of Technology, Hangzhou, 310032 China; 2 School of Petrochemical Engineering and Environment, Zhejiang Ocean University, Zhoushan, 316004 China; 3 Collaborative Innovation Center of Yangtze River Delta Region Green Pharmaceuticals, Zhejiang University of Technology, Hangzhou, 310032 China; 4 Zhejiang Provincial Key Laboratory of Petrochemical Pollution Control, Zhoushan, 316004 China; 5 Zhejiang Zhonglan Environmental Technology Co., Ltd., Wenzhou, 325000 China; 6 Yali High School, No. 428 Laodong Western Road, Changsha, Hunan People’s Republic of China 410007

**Keywords:** VOCs, Magnetic field, Chlorobenzene, Bioaugmentation

## Abstract

**Abstract:**

Magnetic fields (MF) have been proven efficient in bioaugmentation, and the internal MFs have become competitive because they require no configuration, despite their application in waste gas treatment remaining largely unexplored. In this study, we firstly developed an intensity-regulable bioaugmentation with internal MF for gaseous chlorobenzene (CB) treatment with modified packing in batch bioreactors, and the elimination capacity increased by up to 26%, surpassing that of the external MF. Additionally, the microbial affinity to CB and the packing surface was enhanced, which was correlated with the ninefold increased secreted ratio of proteins/polysaccharides, 43% promoted cell surface hydrophobicity, and half reduced zeta potential. Furthermore, the dehydrogenase content was promoted over 3 times, and CB removal steadily increased with the rising intensity indicating enhanced biofilm activity and reduced CB bioimpedance; this was further supported by kinetic analysis, which resulted in improved cell adhesive ability and biological utilisation of CB. The results introduced a novel concept of adjustable magnetic bioaugmentation and provided technical support for industrial waste gas treatments.

**Key points:**

• *Regulable magnetic bioaugmentation was developed to promote 26% chlorobenzene removal*

• *Chlorobenzene mineralisation was enhanced under the magnetic field*

• *Microbial adhesion was promoted through weakening repulsive forces*

**Graphical abstract:**

**Figure:**
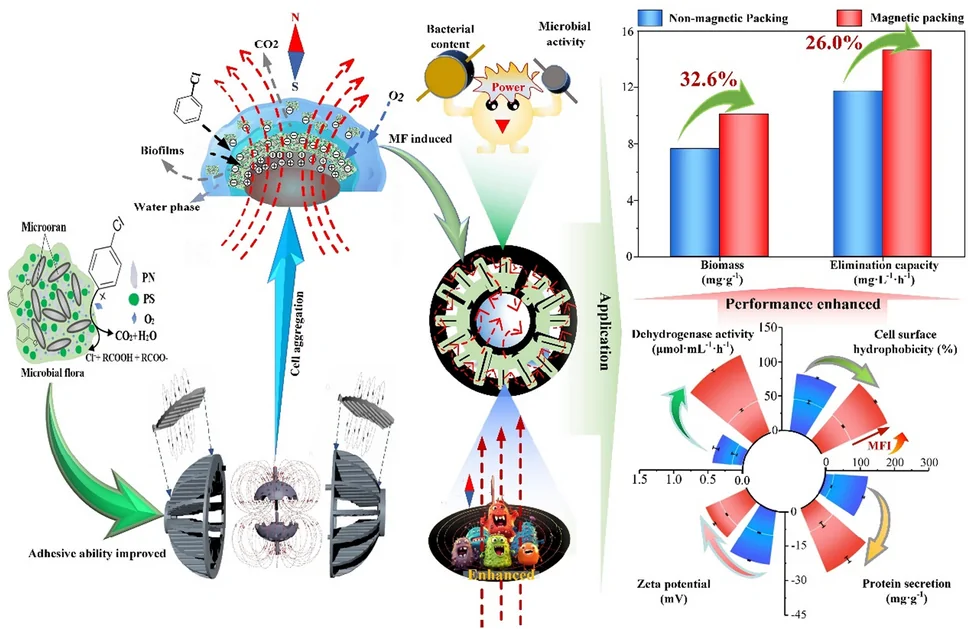

**Supplementary Information:**

The online version contains supplementary material available at 10.1007/s00253-024-13001-z.

## Introduction

The emission of chlorinated volatile organic compounds (CVOCs) showed an increasing trend with the development of the industry, including dyestuffs, pharmaceutical industries, etc. (Lu et al. 2019; Rybarczyk et al. 2019). The CVOC-contaminated gas causes serious environmental damage and threatens human health (Ren et al. 2020). As a typical CVOC, chlorobenzene (CB) has been reported to be a strong cancer-causing agent by the US Department of Health and Human Services, and efficient treatments are urgently required in this severe situation (Zhang et al. 2018). Biofiltration is an efficient and economic treatment for CVOCs with no secondary pollution, but its removal performance was limited by the low water solubility and resistance to degradation. Recently, some researchers have taken various strategies for bioaugmentation and enhanced VOC removal in industrial manufacture; therein, biological technology could play an efficient role in the waste gas treatment of the hydrophobic substances due to the absolute advantage of environmentally friendliness and inexpensiveness (Cheng et al. 2016; Wu et al. 2023). In addition, surfactants could improve the water solubility and fluidity of hydrophobic of insoluble gases, which was evidently suitable for the removal of various n-alkanes in the BTF (Wu et al. 2022), but their removal efficiency are not ideal enough yet. However, the biological impedance of C–Cl bonds limited their biodegradation, and few separated bacterial species demonstrated the ability to utilise the CVOCs efficiently (Béchohra et al. 2016). Besides, the biotoxicity to the bacteria made the biodegradation difficult. Therefore, bioaugmentation is required for similar refractory organic pollutants (Wang et al. 2009; Zhang et al. 2011).

Magnetic field (MF) is proven to have considerable impacts on cell behaviour and microbial activity (Chen et al. 2021). With increased dissolved oxygen concentration, the bacterial growth accelerated due to the promotion of critical enzyme synthesis, and the microbial metabolism strengthened through the enhancement of enzyme activity under MF stimulation simultaneously (Okano 2008). Liang observed increased enzyme secretion and a 1.5-fold promotion of the intracellular lignin peroxidase activity under a 95 mT MF (Tan et al. 2020). Li demonstrated an increased growth rate and shortened generation time under magnetic treatment, which were caused through increasing enzyme activity by 1.5–2.0-folds (Li et al. 2015). Therefore, MF bioaugmentation has tremendous potential for biological waste treatment, and it has been applied in waste water treatment recently. A 19% increase in total nitrogen removal efficiencies was achieved in molasses-containing wastewater treatment under the weak MF (0.9 mT) (Wang et al. 2023). Meanwhile, appropriate magnetic field intensity (MFI) was also explored in the published researches, and a 20–30% increase in RE of phenols, formaldehyde, ammonia, and nitrite was achieved in wastewater treatment under 17.8, 7, and 17 mT MFI, respectively (Filipič et al. 2015; Łebkowska et al. 2013; Yavuz and Çelebi 2000). However, few researches were focus on the MF bioaugmentation in waste gas treatment. Compared with wastewater treatment, the different cell behaviour and characteristics in the multiphase environment of the waste gas treatment probably lead to variations in performances and mechanisms.

Recently, several researches have verified the positive enhancement of the MF in waste gas treatment. Quan demonstrated the bioaugmentation performance with an external MF generator in a trickling biofilter for treating gaseous trichloroethylene and obtained a significant enhancement at an MFI of 60 mT (Quan et al. 2018). However, the processes were achieved under external MF by the MF generator with continuously energy supplement, which was costly.

Packing modification is a typical bioaugmentation process with the advantages of excellent cellular affinity, remarkable effects, and available operation. Various modification methods have been recently developed to optimise moisture retention, microbial attachment, biofilm formation, and the mass transfer process (Ramirez et al. 2009; San-Valero et al. 2017; Wang et al. 2022a, b). As one of the modified packing, magnetic packing with internal MF would be a practical choice owing to its easy operation and no requirement for additional magnetising equipment. In consideration of the strongly oxidic environment in a biological reactor treating waste gas, the difficulties of preventing the magnetic particles from being oxidised and keeping the MF stable are required to be overcome.

Herein, a novel intensity-regulable MF bioaugmentation method with no external magnetic device was developed through the application of magnetic packings for CB biodegradation. The characteristics of the packings, including MFI stability and mechanical strength, were determined. Furthermore, the CB removal performance was investigated, and the mechanisms of the MF bioaugmentation were explored. This study could provide a basis and reference for the industrial applications of CVOC treatment via magnetic bioaugmentation.

## Methods and materials

### Inoculum and culture medium

The CB-degrading bacterium, *Ralstonia* sp. XZW-1 (the strain has been deposited in China Center for Type Culture Collection, and the strain number is CCTCC NO:M 2022557), was previously isolated from the activated sludge of a pharmaceutical factory in Zhejiang province. The mineral medium (MM) contained (per litre of deionised water): 1.0 g of KH_2_PO_4_, 0.20 g of MgSO_4_·7H_2_O, 1.5 g of NH_4_SO_4_, 0.023 g of CaCl_2_, 4.5 g of Na_2_HPO_4_·12H_2_O, and 1 mL of trace element stock solution. The pH of the medium was maintained at 7.0. The trace element stock solution contained (per litre of deionised water) 1.0 g of FeSO_4_·7H_2_O, 0.10 g of MnSO_4_, 0.10 g of ZnSO_4_, 0.02 g of CoSO_4_·6H_2_O, 0.014 g of H_3_BO_3_, and 0.02 g of Na_2_CoO_4_·2H_2_O. The MM was autoclaved using a high-pressure steam steriliser at 110 °C for 40 min, and the conical flasks (250 mL) contained 100 mL of aqueous sample.

### Characterisation of packing

#### Preparation of the packing

**Fig. 1:**
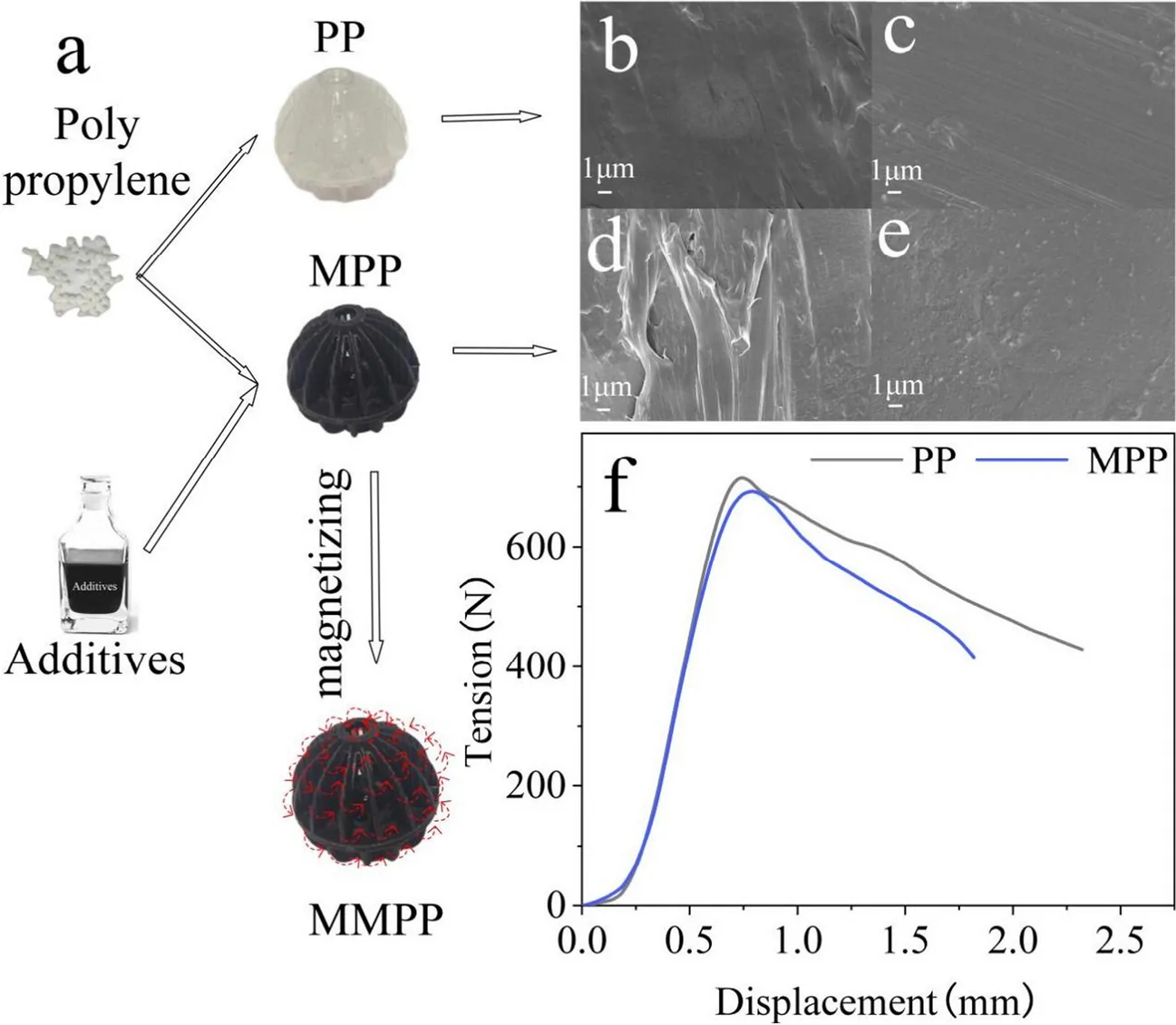
The preparation process of the three packings and their surface morphology and mechanical performance: **a** the production flow chart of the polypropylene packing (PP), the modified polypropylene packings (MPP), and the magnetic-modified polypropylene packing (MMPP); **b**, **c** the picture of the PP through the Scanning electron microscope (SEM); **d**, **e** the picture of the MPP through the SEM; **f** the tension of the PP and MPP under different displacements

The packings were prepared using the extrusion moulding process with a twin-screw injection moulding machine (Zhejiang Shenda Machinery Manufacturing Co., Ltd). Compared to the traditional polypropylene packing (PP), the modified polypropylene packings (MPP) were modified with extra materials including activated carbon, starch, and magnetic powders (Table 1). Similar to the PP, the shape of the MPP is a hollow sphere (Fig. 1) of 30-mm diameter and 1-mm thickness. After being magnetised by the electro-magnetising equipment (Hangzhou Magnetoelectric Technology Machinery Manufacturing Co., Ltd.), the MPP became the magnetic-modified polypropylene packing (MMPP). The properties of the three packings were evaluated in this study.

**Table 1:** Composition and magnetism of the packings

Packing components	PP	MPP	MMPP
PP/%	100	82	82
Magnetic powder/%	0	10	10
Activated carbon/%	0	1	1
White mineral oil/%	0	1	1
Starch/%	0	1	1
Hydroxyapatite/%	0	3	3
Polyvinyl alcohol/%	0	1	1
Magnetism	×	×	√

#### Wettability

The PP, MPP and MMPP were orderly weighed on an electronic scale after being submerged in water for 1, 30, and 14,400 min, respectively, and maintained at 30 °C for 1, 2, 5, 8, 10, 15, 30, 60, 120, 360, 720, and 1440 min while simultaneously weighing and recording the corresponding data.

#### Fourier-transform infrared spectroscopy

The solid samples were first maintained at 80 °C in a vacuum drier for 48 h, then powdered, and subsequently mixed in a ratio of 1:100 with KBr in an agate grinder. Finally, these mixtures were compressed and measured to analyse the major functional groups with a Fourier-transform infrared spectroscopy spectrometer (Nicolet 5700, USA) between 400 and 4000 cm^−1^.

#### Magnetic field intensity

The magnetic field intensity was detected by Gauss meter (KT-101) after 5 min preheat and test range setting with tightly locked.

### Characterisation analysis

#### Batch experiments

Biodegradation experiments were conducted in 250-mL conical flasks containing 100 mL of sterile MM in a rotary shaker (160 rpm) at 30 °C. To ensure the same initial biomass concentration, 1 mL of *Ralstonia* sp*.* XZW-1 (OD_600_ = 1.0) suspension was added to every conical flask to ensure a constant initial CB concentration. The headspace gas of the flasks was collected at indicated intervals, and the aqueous samples were collected after the biodegradation for further analysis.

The impacts of internal MF (from the MMPP) and external MF (from the magnet blocks outside the flasks) were both explored in this study. The internal MFI was regulated by adjusting the magnetic packing proportion in the conical flask. Meanwhile, the influences on the biodegradation of the external MF were also evaluated whose MFI was controlled by moving the magnet blocks outside the flasks.

#### Surface morphology

The surface morphology of the sample was identified using a scanning electron microscope (SEM SU8010, Japan), the hydrophilicity was characterised using a contact angle instrument (JY-82B Kruss DSA), and the mechanical properties were characterised by a universal material testing machine (CMT-6104 China).

#### Gaseous chlorobenzene concentration and CO_2_ production determination

The gaseous concentration of CB was evaluated using GC (Agilent 6890, USA). The temperatures of the inlet, column (HP-Innowax), and hydrogen flame ionisation detector were 250 °C, 200 °C, and 280 °C, respectively. The liquid concentration of CB was calculated using the gas–liquid partition coefficient from the gas concentration of CB according to our previous work (Chen et al. 2018). An Agilent 6890 GC (Santa Clara, CA, USA) was used to measure the concentrations of CO_2_, and the temperatures of the inlet, column (HP-Plot-Q, 30 m × 320 µm × 20 µm), and thermal conductivity detector were 100 °C, 40 °C, and 180 °C, respectively.

#### Chloride concentration

The aqueous samples were first filtered using a 0.45-μm filter membrane and sodium cation exchange column. The chloride concentration was detected using ICS-2000 ion chromatography. The separation column used was AS19 (4.0 × 250 mm), the temperature of the conductivity detector was 35 °C, the drenching solution was KOH (concentration gradient 10–40 mmol·L^−1^), the flow rate was 1.00 mL·min^−1^, and the injection volume was 25 μL.

### Chemical characterisation

#### EPS content

The EPS was mainly composed of polysaccharides (PS) and proteins (PN). The PS was extracted and quantified using high-temperature treatment and anthrone-sulfuric acid colorimetry, and the protein content was measured using the Bradford method (Bradford 1976). The data were obtained according to the standard curve.

#### Cell surface hydrophobicity

The cell surface hydrophobicity (CSH) of the bacteria was calculated according to the characteristics of bacterial adhesion to hydrocarbons (Rosenberg and Kjelleberg 1986). The absorbance of the aqueous phase (AP) was expressed by OD_600_, and the percentage of adhesion to the packing was calculated using the following equation.

1 $$\mathrm{CSH\;of\;the\;cell}=\left(1-\mathrm{the\;absorbance\;of\;AP}/\mathrm{the\;absorbance\;of\;the\;cell\;suspension}\right)\times 100\%.$$

#### Biomass

The attached biomass was represented by the cell dry biomass, which was dried at 105 °C until constant weight, and the suspended biomass was represented by the OD_600_ value, which was measured using a spectrophotometer at 600 nm.

#### Zeta potentials

The zeta potentials were detected by the BeNano 90 Zeta nanometer potential analyzer after 100 runs at 25℃ with an interval of 30 s (Mohammadi-Jam et al. 2022), and the results were the average of three samples.

#### Dehydrogenase activity

The aqueous sample (0.3 mL), tris–HCl buffer (pH = 7.5, 1.5 mL), and iodine–nitro tetrazolium violet solution (0.2%, 1 mL) were orderly mixed in a 10-mL centrifuge tube. The prepared samples were quickly incubated in a 37 °C water bath with constant shaking for 30 min. Subsequently, 1 mL of analytically pure formaldehyde was added at a concentration of 37% to terminate the enzyme reaction, and the dehydrogenase activity (DHA) was measured by following the standard curve.

#### Bacterial attachment on the packing

The aqueous samples (100 mL) with varying OD_600_ were prepared in 310-mL sealed bottles. The PP, MPP, and MMPP were simultaneously added to the bottles. Followed by vigorous shaking for 5 min and being left to stand for 30 min, the attached and suspended biomass were measured.

#### Three-dimensional excitation–emission matrix fluorescence spectra of EPS

The three-dimensional excitation–emission matrix fluorescence spectra were measured using a Shimadzu RF-6000 spectrofluorophotometer (Horiba, Japan), and the EPS samples without dilutions were scanned over excitation wavelengths from 245 to 400 nm at 5 nm sampling intervals, and emission spectra were obtained for every 5 nm from 300 to 550 nm. The excitation and emission slit bandwidths were set to 5 nm.

#### Influence of MFI on the magnetic packing

The influence of the MFI on the CB removal and microbial characteristics was explored with the control of the magnetic packing proportion on both magnetic and non-magnetic packings, and the details are shown in Table 2. Owing to the stable average MFI of the MMPP at 1.8 mT (1.0–4.0 mT), the proportion of the magnetic packings (listed above) can be used to control the total MFI in the bio-system.

**Table 2:** The magnetic packing proportion regulation

Magnetic packing	Magnetic proportion (%)	MFI/mT
0	0	0
1	20	1.8
2	40	3.6
3	60	5.4
4	80	7.2
5	100	9.0

The values of magnetic field intensity (MFI) were reference values calculated after the determination of the packing’s average MFI (1.8 mT/packing approximately)

#### Bioreaction kinetics

To study the degradation kinetics of different packings, a series of batch experiments were conducted under different initial concentrations, and the CB biodegradation and growth kinetics were followed the Haldane model, while the decay kinetics were followed the Monod models (Saravanan et al. 2008).

2 $$\mu =\frac{1}{X}\frac{dX}{dt}=\frac{{\mu }_{{\mathrm{max}}}S}{{K}_{S}+S+{S}^{2}/{K}_{I}}$$

3 $$v=\frac{1}{X}\frac{dS}{dt}=\frac{{v}_{{\mathrm{max}}}S}{{K}_{S}+S+{S}^{2}/{K}_{I}}$$

4 $$\frac{dX}{dt}=-{k}_{d}X$$

The parameters *K*_s_, *μ*_max_, *K*_I_, and *v*_max_ represent the half-saturation constant, maximum specific growth rate of the microorganisms and substrate inhibition constant, and maximum specific degradation rate of the CB, respectively. Besides, the *X*, *S* and *K*_d_ were the biomass concentration, CB concentration, and decay coefficient, respectively.

## Results

### Physical morphology

#### Morphology and mechanical performance

The PP and the MPP were both obtained under a continuous manufacturing process under similar conditions, including the same temperature and formulation components (Table 1). A smooth surface was observed on the PP, which enhanced the fluidity in the liquid–gas interface (Shi et al. 2023) and reduced the bacterial adhesive ability. A rough surface with sufficient grooves and microscopic pores was observed for the MPP, which could be attributed to heterochromatic particles in their cross section. The additives created a rough surface and provided a considerable medium for bacterial growth.

The distinctive differences in the structural force between the PP and MPP were compared, and the results are shown in Fig. 1. As the compressive strength increased from 0 to 600 N, the values of displacement ranged between 0 and 0.8 mm. The results indicated that the ductility of the MPP was higher than that of the PP. The effect of physical strength on the displacement was gradually evident until the strength exceeded 600 N, and the maximum tolerable value of the MPP packing was smaller but similar to the PP (Injorhor et al. 2022). The results proved that the packings can meet the mechanical strength requirements of industrial applications (Han et al. 2016).

#### Wettability, hydrophobicity and magnetism

**Fig. 2:**
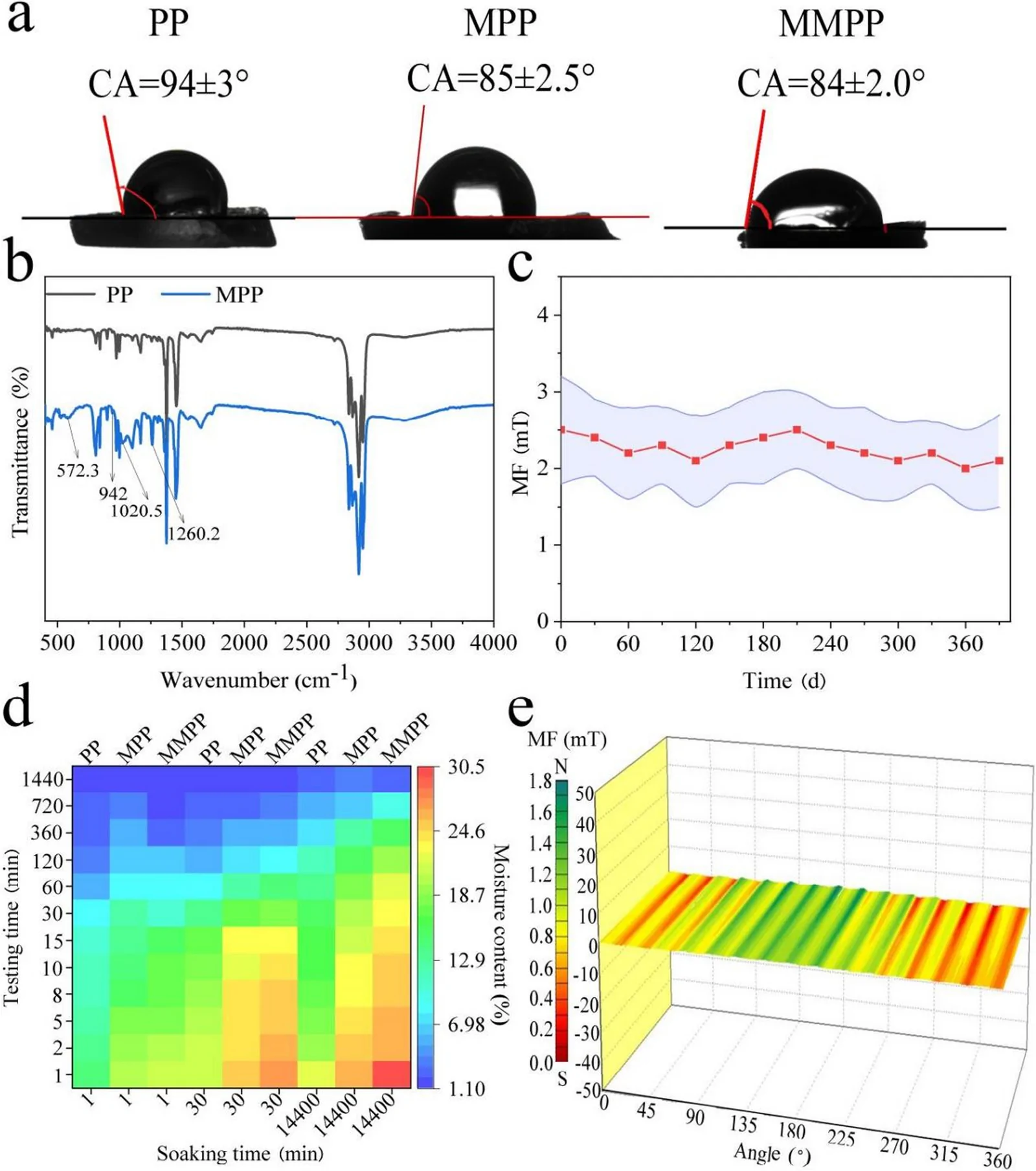
The characteristics of the packings: **a** the contact angle of the three packings; **b** the Infrared spectrogram of the PP and MPP; **c** the magnetic field intensity (MFI) stability of the MMPP over time; **d** the water content of the three packings under different soaking time; **e** the MFI distribution around the MMPP

The characteristics of the packings were measured including the wettability, hydrophobicity and magnetism (Fig. 2). After modified, the contact angles decreased to 85° on the surface of the MPP, compared to the value of 94° for the PP. The results indicated that the surface hydrophobicity decreased after modification craft. The water storage proportion of the MPP was 25.85%, when the soaking time was 14,400 min, which was 1.14 times that of the PP (22.62%). Fe–O and Si–OH bonds were observed at 572.3 and 942.0 cm^−1^ on the surface of the MPP, while only polypropylene was observed in PP (Dutta et al. 2018). The results revealed that the packings were successfully modified. Due to the presence of the skeletal vibration, the magnetic powder was incorporated into the polypropylene substrate, with peaks of Si–O in hydroxy apatite, near 1260.2 and 1020.5 cm^−1^ (Chen et al. 2011).

After magnetised, the MMPP achieved the highest water storage proportion of 30.48% and the contact angle decreased to 84°. Polyvinyl alcohol reduced the packing surface tension, and the MF produced by magnetic powders changed the packing water-holding capacity and strengthened the osmotic pressure of water (Zieliński et al. 2018). Thus, the modification and magnetising process promoted the hydrophilicity and water storage.

The MF distribution analysis revealed that over 75% of the surface was magnetised. Therein, 25% of the surface had an MFI of 1.0–1.6 mT, and the remaining 50% had an MFI of 0.7–1.0 mT. As a hollow spherical structure, the MFI ranging from 0.5 to 2.0 mT was detected around the spherical packing, which was stable between 1.0 and 4.0 mT in the interiors. Besides, the stability was verified, and the MFI was stable after 390 days.

#### Performance evaluation

**Fig. 3:**
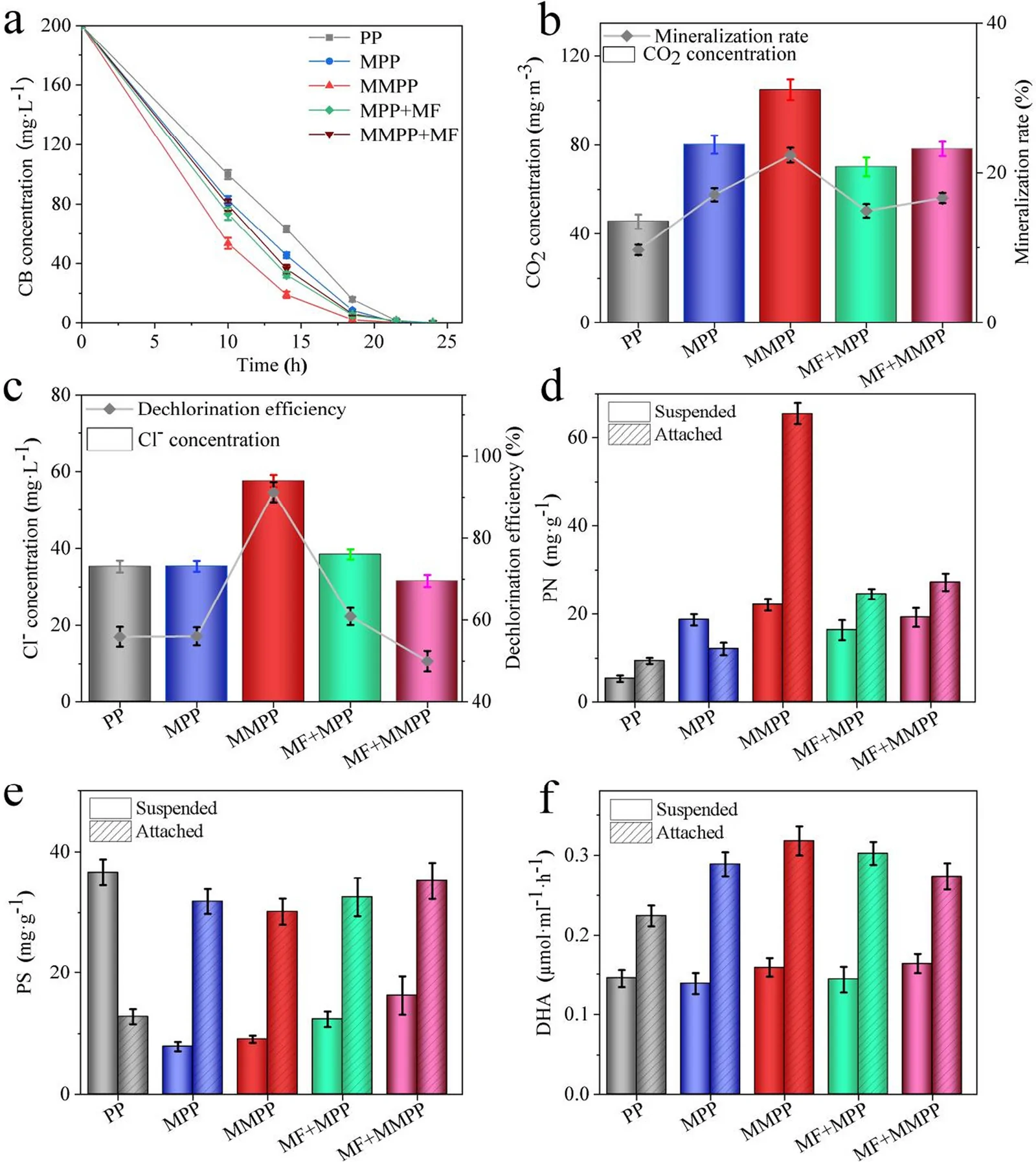
The comparison of the degradation properties in five situations, including the PP only (PP), the MPP only (MPP), the MMPP only (MMPP), the MPP with additional external magnetic field (MPP + MF), and the MMPP with additional external magnetic field (MMPP + MF): **a** the concentration of the chlorobenzene (CB); **b** the concentration of CO_2_ produced by the microorganisms and the mineralisation rate; **c** the concentration of the Cl^−^ and the dichlorination effeciency; **d**–**f** the extracellular protein(PN) secretion, the extracellular polysaccharide (PS) secretion, and the dehydrogenase activity (DHA) of the suspended and attached (to the packings) microorganisms

The MMPP achieved the best degradation performance to eliminate CB (200 mg·L^−1^) in 10.0 h of 14.7 mg·L^−1^·h^−1^, while the removal rate was 11.7 and 10.0 mg·L^−1^·h^−1^ in the groups added with the MPP and PP, respectively (Fig. 3a). Besides, compared the MPP with external MF to the MPP, the removal performance of the internal MF still had an advantage of 8%. The results indicated the positive influence of the internal MF on the biodegradation performance. Surprisingly, the performance declined by 4% when an external MF was superimposed to the internal MF, which probably because of the biological inhibitions caused by the antagonism between the two MFs. For further exploration, the MMPP was used in a bio-trickling filter treating CB, and an average removal efficiencies enhancement of 26.6% was observed compared to the MPP (Fig. S1). The results proved the promotion of CB removal under the internal MF through the MMPP. The CO_2_ and Cl^−^ emissions agreed with the degradation performance in the five groups, with the highest results in the MMPP group.

Similarly, the highest PN secretion and dehydrogenase activity (DHA) were observed with internal MF only, while the lowest PS concentration was observed there (Fig. 3d–f). These results showed high similarity to Yuan’s conclusion (Yuan and Wang 2013), and the increased PN/PS ratio was benefit to the biofilm formation. Additionally, Lebkowska (Łebkowska et al. 2018) demonstrated that the DHA of activated sludge was positively influenced by a 7.5 mT MF with a 25% promotion of COD removal. Besides, the DHA values of the attached cells were much higher than the suspended ones. Therefore, the results proved that MF can stimulate bacterial behaviour, which might benefit biofilm formation and CB elimination.

#### Cell accumulation

**Fig. 4:**
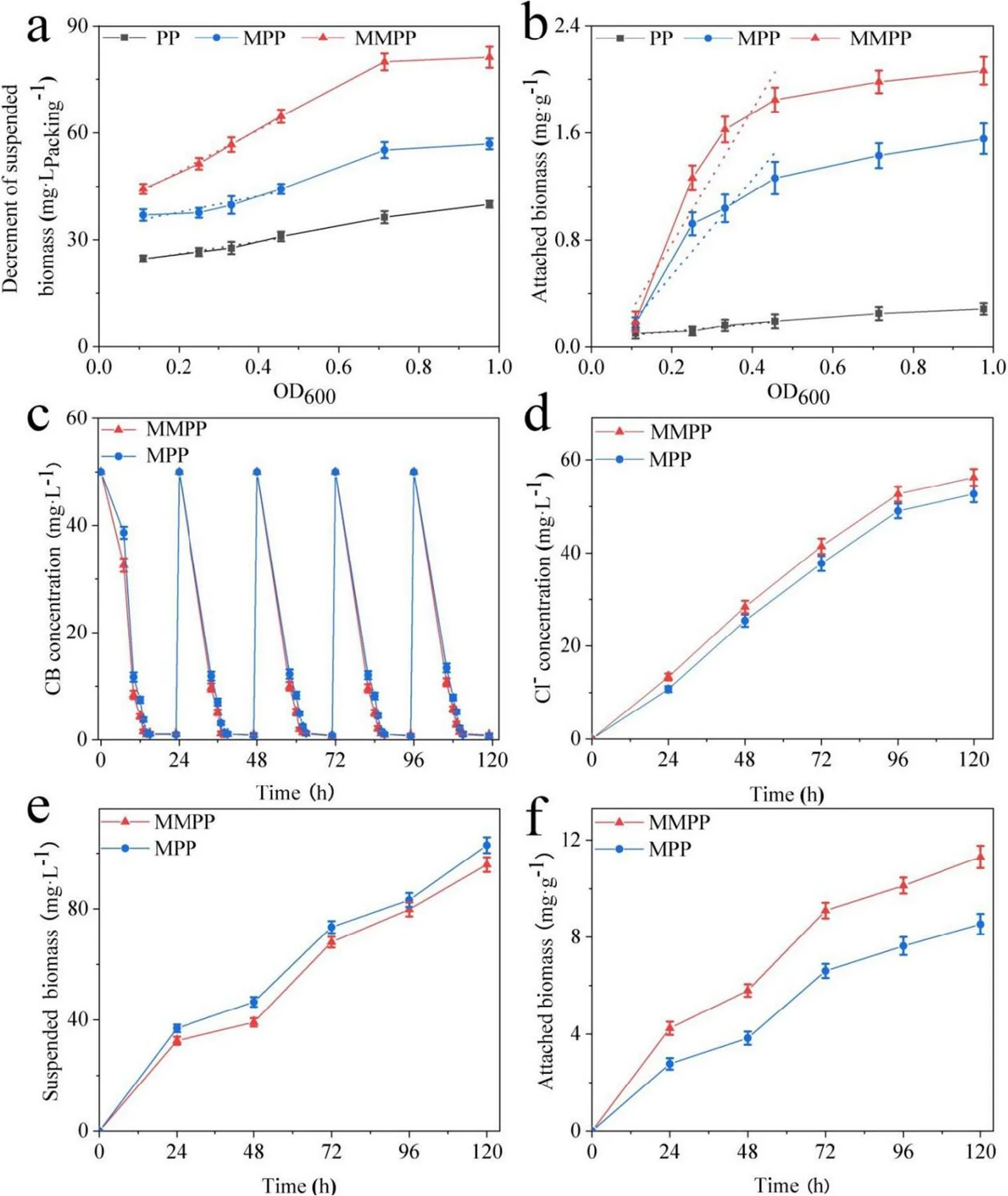
Microbial characteristics of the packings: **a**, **b** the decrement of the suspended biomass in the aqueous phase with the three packings; **b** the increment of the attached biomass on the three packings; **c**–**f** the chlorobenzene concentration, the Cl^−^ concentration, the biomass in the aqueous phase, and the biomass accumulated on the packing surface the in sequential batch experiment

The suspended biomass decrement and attached biomass increment of all the packings maintained an increasing trend under the initial OD_600_ of 0.110. It illustrated the bacterial tendency to adhere to the packing surface. As mentioned previously, the wettability and hydrophobicity of the MPP were optimised for the microbial adhesion by the additives, the modified packings attracted more cells (Sennour et al. 2009). The MMPP group achieved maximal suspended biomass decrement (81.2 mg·L^−1^) and attached biomass increment (2.07 mg·g_packing_^−1^), which was evidently higher than that of the MPP (56.9 mg·L^−1^/1.56 mg·g_packing_^−1^) and the PP group (40.0 mg·L^−1^/0.284 mg·g_packing_^−1^), respectively (Fig. 4a, b). The results indicated that the MF enhanced the adhesion tendency. In the enhancement of the positive charge on the surface of the MMPP caused by the changes of the mixed liquor colloid under the MF, the MMPP became more attractive to the microbes with negative cytomembrane than the MPP and the PP (Ma et al. 2021). Subsequently, sequential batch degradation of the MMPP and MPP was operated, and increasing trends of Cl^−^ as well as attached and suspended biomass accumulation were observed (Fig. 4c–f). The CB was eliminated in 24 h all the batches. Higher concentration of Cl^−^ was observed in the MMPP, compared to the others, throughout the experiment. The results probably provided an information that MF activated the bacteria and promoted the complete mineralisation of CB, which was observed from the CB conversation rate to Cl^−^ (Kraakman et al. 2011). Besides, less suspended biomass was observed in the MMPP, compared to the others, when more attached biomass accumulation was there. It further proved the enhancement of the MF on the bacterial adhesion to the packings (Moore 1979). Related to the result obtained previously that higher DHA of the attached cells, compared to the suspended ones, the MF strengthen the overall activity of microorganisms, which was benefit for CB biodegradation (Wang et al. 2022a, b).

### Effect of MFI on biodegradation and microbial characteristics

#### Biodegradation performance

**Fig. 5:**
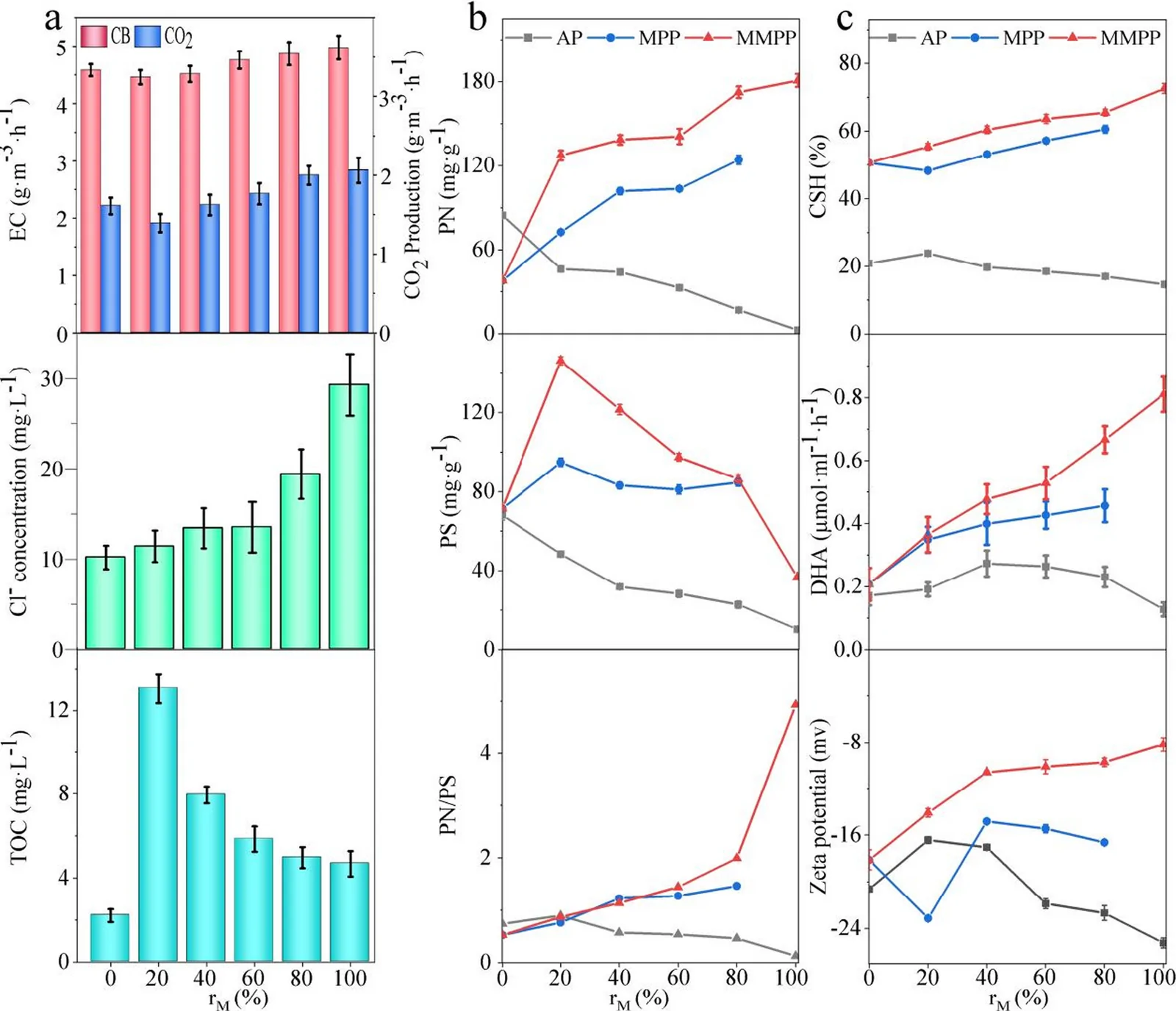
Degradability and cellular characteristics of the bacteria under the different MFI: **a** the elimination capacity and the CO_2_ production, the Cl^−^ concentration, and the total organic carbon (TOC) in the aqueous phase; **b** the extracellular protein (PN) secretion, the extracellular polysaccharide (PS) secretion, and the ratio between the PN and PS (PN/PS); **c** the cell surface hydrophobicity (CSH), the dehydrogenase activity (DHA), and zeta potential of the cellular surface

The variations in biodegradation under different MFIs with regulating the proportion of the MMPP among the packings were also explored in this study (Fig. 5a). With the increasing MFI (the proportion of the MMPP among the packings, r_M_), the CB removal rate, CO_2_ production, and Cl^−^ accumulation gradually increased, and the highest values were 4.98 mg·L^−1^·h^−1^, 2.06 mg·L^−1^·h^−1^, and 29.3 mg·L^−1^, respectively. Except for the initial point, the total organic carbon (TOC) showed a decreasing trend in the aqueous phase; the lowest value was 4.68 mg·L^−1^ when the r_M_ was 100%.

The results demonstrated a gradually strengthened positive influence on CB biodegradation and mineralisation under increasing MFI. However, it has been reported that the strengthened MF (> 130 mT) had a negative influence on the micro-organisms. Hence (Quan et al. 2017), an optimum value of MFI for magnetic bioaugmentation needs to be established in further studies. In consideration of the fact that the MFI can be regulated by adjusting the r_M_, the results offer important technical support for intensity regulatable magnetic bioaugmentation in the industrial VOCs contaminated emission treatments.

#### Bacterial characteristics under variation MFI

In order to further evaluate the feasibility of the intensity regulatable magnetic bioaugmentation through adjusting the r_M_, the bacterial characteristics under variation MFI was explored (Fig. 5b). With the increase of the r_M_, the MFI in the flasks increased, and the PN content gradually was observed to raise from 37.9 to 180.8 and 124.2 mg·g^−1^ on the surface of the MMPP and the MF-induced MPP, respectively. In contrast, the PN content gradually decreased from 84.5 to 2.56 mg·g^−1^ in the aqueous phase (AP). The results indicated that the MF stimulated the bacterial PN secretion and induced the PN adhesion to the packing surface simultaneously (Wang et al. 2018). Furthermore, a declining trend was observed for the PS on the MMPP surface, and in the AP, while it remained similar in the MF-induced MPP. This revealed that the MF could influence the PS secretion and utilisation, but the effects weakened with distance limitation on the MF-induced MPP (Donot et al. 2012). Besides, more PN and PS content were observed on the packings than in the suspensions, and it was probably benefit to the biofilm formation on the packing surface because the EPS (the PN and PS) was the main component of the biofilm (Liu et al. 2018). The values of PN/PS increased significantly to 4.93 with the increasing r_M_ and the strengthening of MFI in the MMPP, and an increment was also observed in the MF-induced MPP. The microbial EPS secretion, which is strongly connected with the CSH, microbial adhesion, and flocculation, has a considerable influence on cell adhesion and biofilm formation (You et al. 2023). Furthermore, the achievements of the tight adhesion between the cells and packing surface were also reported through the microbial EPS secretion under the MF.

The CSH gradually increased from 50.6 to 72.6% and 60.6% with strengthening MF in the MMPP and the MF-induced MPP (Fig. 5c), respectively. It was closely related to the increasing PN secretion, which belongs to the hydrophobic part of EPS in the biofilm with the non-polar groups (Zhang et al. 2015). The cells with a higher CSH demonstrated a better biodegradability degradation rate because of the easy adsorption of hydrophobic CB molecules (Zhang et al. 2010). The zeta potential also increased from − 16.6 to − 8.20 mV in the MMPP, but it fluctuated between − 23.1 and − 14.8 mV in the MF-induced MPP. The positively charged PN neutralised the negatively charged cytomembrane (Liao et al. 2001), enhancing the bacterial aggregation on the packing by reducing the repulsive Coulomb force between the bacterial cells (Zikmanis et al. 2007). Furthermore, the increasing trend of DHA from 0.207 to 0.810 and 0.457 µmol·ml^−1^·h^−1^ on the surface of the MMPP and the MF-induced MPP, respectively, demonstrated the promotion of microbial activity under strengthening MFI (Ren et al. 2019). Therefore, the CB removal performance was expected to increase with the promotion of the microbial CB affinity and activity. However, an opposite trend was observed in the AP, probably due to the exfoliation of the dead bacteria with poor dehydrogenase from the biofilm (Yang et al. 2018).

The proteoid substances (IV) represent the microbial metabolism when the humic acid (V) represents the microbial apoptosis in the three-dimensional excitation–emission matrix fluorescence spectra. An increasing area of IV was observed in the MMPP and the MF-induced MPP (Fig. S2). It illustrated that the microbial metabolism strengthened with the increasing MFI. Besides, the area of V was smaller in the samples from the MMPP and the MF-induced MPP compared to the microorganisms in the AP. This phenomenon was consistent with the results of the DHA variation under different MFI.

As a conclusion, the r_M_ can influence the bacterial characteristics through regulating the MFI. The biofilm formation, microbial CB affinity, and microbial activity were enhanced with the raising MFI under 9.0 mT. The results provide strong evidence of the feasibility of the intensity regulable magnetic bioaugmentation by changing the proportion of the magnetised packings.

#### Biodegradation kinetics

**Fig. 6:**
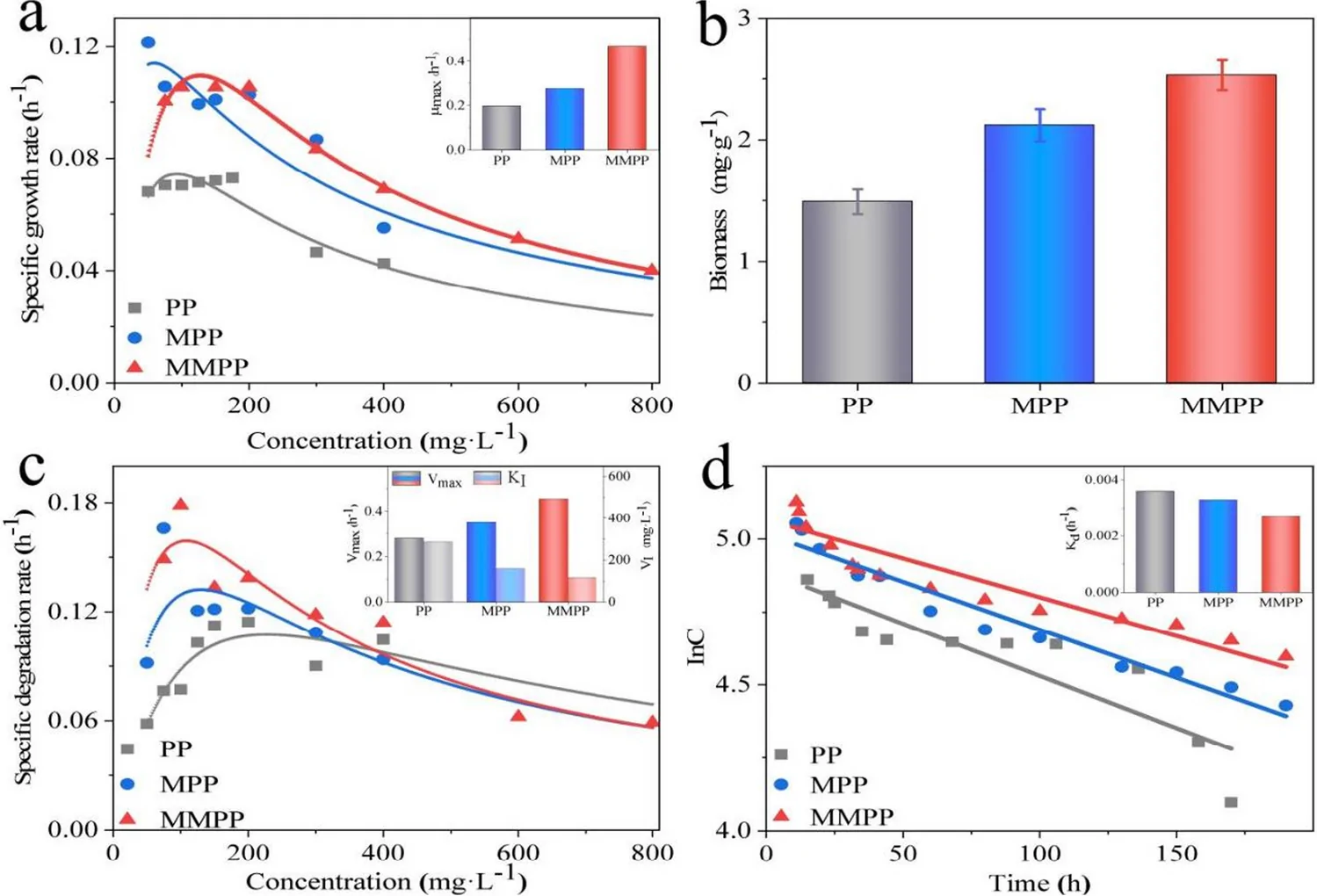
Microbial growth and biodegradation kinetics for the three packings: **a** the fitting curve of the growth kinetics and the specific growth rate (*μ*_max_), **b** the attached biomass which were detected experimentally, **c** the fitting curve of the biodegradation kinetics, the specific biodegradation rate (*v*_max_) and the substrate inhibition constant (*K*_I_), **d** the fitting curve of the decay kinetics and the decay constant (*K*_d_)

The specific growth curve displayed the sequencing of the specific growth rate of the micro-organism (µ) as *µ*_MMPP_ > *µ*_MPP_ > *µ*_PP_, and the maximum specific growth rate (µ_max_) was 0.470, 0.276, and 0.197 for MMPP, MPP, and PP, respectively (Fig. 6 and Table S1). The experimental results for biomass were in good agreement with the model predictions (Fig. S3). Similarly, the specific degradation curve revealed the highest value of the maximum specific biodegradation rate (*V*_max_) of MMPP (0.455), which was followed by MPP (0.350) and PP (0.279). Inversely, the inhibition constant (*K*_I_) and decay constant (*K*_d_) were sequenced in the opposite order: PP > MPP > MMPP (284.7 > 157.9 > 115.2 and 0.0036 > 0.0033 > 0.0027 h^−1^). The decay constant of the MMPP group was lower than that of the other groups, which was in accordance with the increased microbial activity under the MF. The results indicated that the promotion of CB degradation was based on activated microbial growth and biodegradation as well as the weakened inhibitory effect of CB (Dorado et al. 2015).

## Discussions

The biofilm played a core role in the CB removal, and the biofilm formation starts from the initial adhesion. Basis on the results, we found that the enhanced biofilm formation was mainly related to two causes related to the characteristics of the packing surface and the bacteria respectively. Firstly, the process of packing modification strengthened the affinity between the cell and the packing surface, through introducing additives with hydrophilic groups, like starch and activated carbon, which changed the characteristics of the packing surface. Usually, the PP has limited affinity with organisms as a smooth hollow sphere through injection mould, which had poor compatibility with inorganic packings, polar conducts, and reinforcing substrates, and belonged to non-polar thermoplastic polymers with certain crystallinity (Aboubakri et al. 2022). After the modification, the MPP showed considerable bio-affinity and hydrophilicity because of the hydrophilic additives.

Secondly, the MF positively influences the biofilm formation process as well through reducing the negative charge of the cytomembrane and change the cellular behaviour. The effect of the negative charge reducing is twofold owing to the modified packing surface which is usually positively charged (Zhou et al. 2010). On the one hand, the Coulomb force between the cytomembrane and the packing surface was weakened, which probably limited the cellular adhesion; on the other hand, owing to the electriferous groups, they were neutralised in the EPS by amino acid protein with positive charge, and it directly decreased the electrostatic repulsion of bacterium cell. In addition, the inhibition of electrostatic repulsion increased cell adhesion, and more cellular adhesion were allowed during the neutralization, which was benefit to biofilm formation (Hayashi et al. 2001; Vandana et al. 2023). Our experimental results illustrated that the latter played a dominant role and its positive effects covered the negative ones from the weakening Coulomb force. Similar conclusions were also found in previous publications (Nadell et al. 2015). Besides, the changes of the cellular behaviour, especially the EPS secretion, influence the affinity between the biofilm and the packing surface. The improved PN secretion increased the cell surface hydrophobicity (Mahto et al. 2022), which led to the enhanced affinity between the bacterial cells and the packing surface through promoting the similarity of hydrophobicity. Meanwhile, the increasing EPS secretion helps the bacterial cells to form stable three-dimensional spatial structures, which were benefit to the adhesion stability of biofilm. Therefore, the modification process lays the foundation for the removal enhancement.

Recently, a primary difficulty of external MF was the oxidisation of the magnetic compounds; Fe_3_O_4_ is easy to transform to Fe_2_O_3_ in humid environment over a pH range of 5–11 (Oliveira et al. 2002). It was solved through the protection of the polypropylene, which fixed the magnetic component inside and isolated it from the erosion of oxygen. The stable magnetic field can continuously affect the microbial degradation process.

Totally, this study proved that the magnetic field have a positive influence on the degradation performance of microorganisms, and in fact, various species of microorganisms are greatly affected by different magnetic field strength.

In addition to enhancing biodegradation, magnetic fields promoted extra functions through affecting the cellular behaviour and metabolic processes. The start-up shortening and bio-H_2_ production enhancement were found, and the optional MFI was 70 mT for the *Clostridium*, *Lactobacillus*, and *Terrisporobacter* dominant microbial community (Arriaga et al. 2023).

After the degradation, the MMPP achieved the highest mineralisation rate of 22.34%, while the left was transferred into the biomass and intermediate product, and 91.23% of the element Cl was transferred into the chloridion. The results also indicated that the MF promoted the mineralisation of the CB.

**Fig. 7:**
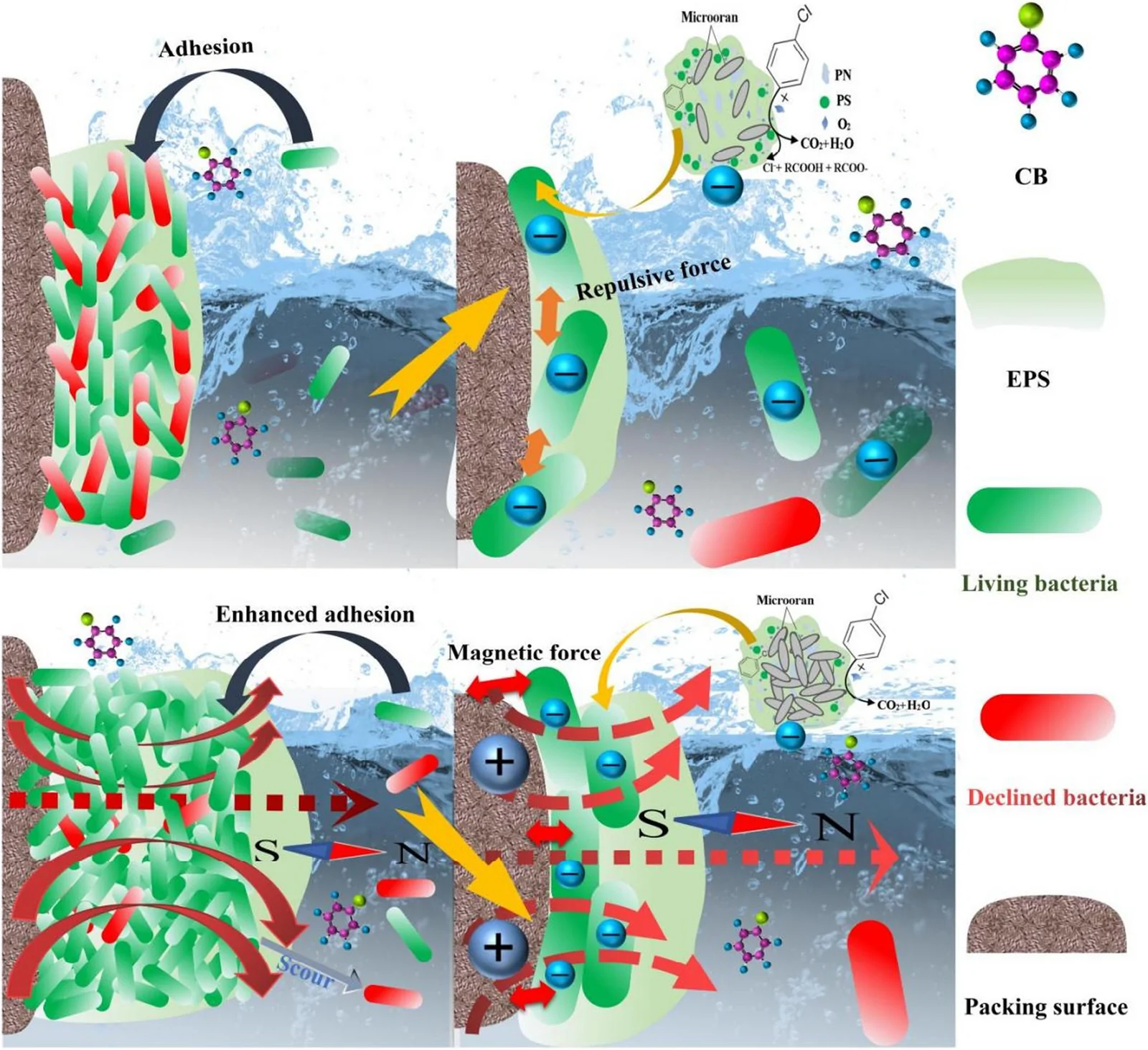
Diagrammatic mechanism of chlorobenzene biodegradation enhancement under the magnetic field

Basis on the results from the biodegradation process, the magnetic strengthening mechanism can be concluded. The MMPP affected the entire CB biological removal process, including biofilm formation and biodegradation (Fig. 7). The MMPP, having a rough and positively charged surface with varying porosity (Rakoczy et al. 2017), was suitable for cell adhesion. The altered EPS secretion and CSH in the MF promoted the affinity between the cytomembrane and the packing surface. Thus, the bacterial cells adhered easily to the surface of the MMPP. The biofilm formation was enhanced through the reduction of repulsion force between the cells owing to the neutralised negatively charged cytomembrane. Furthermore, the enhanced microbial growth (biomass accumulation and the specific growth rate) also accelerated biofilm formation. In consideration of the biodegradation process, the microbial CB affinity was improved with the altered EPS secretion and CSH in the MF. With the weakened biological inhibition of CB (the inhibition constant) and the increased biological metabolic capacity (DHA), CB removal was consequently enhanced.

As a conclusion, intensity-regulable and stable magnetic bioaugmentation with no extra equipment was developed for the gaseous CB treatment initiatively with an innovative magnetically modified PP packing. It promoted the cellular adhesive tendency through optimising the ratio of proteins/polysaccharides, cell surface hydrophobicity, and zeta potential, which accelerated the biofilm formation. Besides, the advanced biological metabolic capacity and alleviated biological inhibition of CB was also observed under the MF, which consequently led to the enhancement of CB removal. This study provided a novel concept of regulable magnetic bioaugmentation and provided technical support for the industrial gaseous CVOC treatment.

## Supplementary Information

Below is the link to the electronic supplementary material.

Supplementary file1 (PDF 567 KB)

## Data Availability

Data and materials will be made available on reasonable request.

## References

[CR1] Aboubakri A, Akkus Y, Sadaghiani AK, Sefiane K, Koşar A (2022) Computational and experimental investigations on the evaporation of single and multiple elongated droplets. Chem Eng J Adv 10:100255. 10.1016/j.ceja.2022.100255

[CR2] Arriaga S, Carboni MF, Lens PNL (2023) Effect of static magnetic field exposure on biohydrogen production via dark fermentation of glucose. Process Saf Environ 176:375–388. 10.1016/j.psep.2023.06.022

[CR3] Béchohra I, Le Menn JB, Couvert A, Amrane A (2016) Activated sludge acclimation for toluene and DEHP degradation in a two-phase partitioning bioreactor: biodegradation of volatile organic compounds by activated sludge. Int J Environ Sci Technol 13:1883–1890. 10.1007/s13762-016-1019-y

[CR4] Bradford MM (1976) A rapid and sensitive method for the quantitation of microgram quantities of protein utilizing the principle of protein-dye binding. Anal Biochem 72:248–254. 10.1006/abio.1976.9999 942051 10.1016/0003-2697(76)90527-3

[CR5] Chen HJ, Wang YM, Qu JM, Hong RY, Li HZ (2011) Preparation and characterization of silicon oil based ferrofluid. Appl Surf Sci 257:10802–10807. 10.1016/j.apsusc.2011.07.103

[CR6] Chen D-Z, Zhao X-Y, Miao X-P, Chen J, Ye J-X, Cheng Z-W, Zhang S-H, Chen J-M (2018) A solid composite microbial inoculant for the simultaneous removal of volatile organic sulfide compounds: preparation, characterization, and its bioaugmentation of a biotrickling filter. J Hazard Mater 342:589–596. 10.1016/j.jhazmat.2017.08.079 28892796 10.1016/j.jhazmat.2017.08.079

[CR7] Chen L, Zhang K, Wang M, Zhang Z, Feng Y (2021) Enhancement of magnetic field on fermentative hydrogen production by Clostridium pasteurianum. Bioresource Technol 341:125764. 10.1016/j.biortech.2021.125764 10.1016/j.biortech.2021.125764 34438289

[CR8] Cheng Y, He H, Yang C, Zeng G, Li X, Chen H, Yu G (2016) Challenges and solutions for biofiltration of hydrophobic volatile organic compounds. Biotechnol Adv 34:1091–1102. 10.1016/j.biotechadv.2016.06.007 27374790 10.1016/j.biotechadv.2016.06.007

[CR9] Donot F, Fontana A, Baccou JC, Schorr-Galindo S (2012) Microbial exopolysaccharides: main examples of synthesis, excretion, genetics and extraction. Carbohyd Polym 87:951–962. 10.1016/j.carbpol.2011.08.083

[CR10] Dorado AD, Dumont E, Muñoz R, Quijano G (2015) A novel mathematical approach for the understanding and optimization of two-phase partitioning bioreactors devoted to air pollution control. Chem Eng J 263:239–248. 10.1016/j.cej.2014.11.014

[CR11] Dutta B, Shetake NG, Gawali SL, Barick BK, Barick KC, Babu PD, Pandey BN, Priyadarsini KI, Hassan PA (2018) PEG mediated shape-selective synthesis of cubic Fe_3_O_4_ nanoparticles for cancer therapeutics. J Alloy Compd 737:347–355. 10.1016/j.jallcom.2017.12.028

[CR12] Filipič J, Kraigher B, Tepuš B, Kokol V, Mandić-Mulec I (2015) Effect of low-density static magnetic field on the oxidation of ammonium by Nitrosomonas europaea and by activated sludge in municipal wastewater. Food Technol Biotechnol 53:201–206. 10.17113/ftb.53.02.15.3629 27904349 10.17113/ftb.53.02.15.3629 PMC5068407

[CR13] Han L, Shaobin H, Zhendong W, Pengfei C, Yongqing Z (2016) Performance of a new suspended filler biofilter for removal of nitrogen oxides under thermophilic conditions and microbial community analysis. Sci Total Environ 562:533–541. 10.1016/j.scitotenv.2016.04.084 27110967 10.1016/j.scitotenv.2016.04.084

[CR14] Hayashi H, Tsuneda S, Hirata A, Sasaki H (2001) Soft particle analysis of bacterial cells and its interpretation of cell adhesion behaviors in terms of DLVO theory. Colloids Surf, B 22:149–157. 10.1016/S0927-7765(01)00161-8 10.1016/s0927-7765(01)00161-8 11451661

[CR15] Injorhor P, Trongsatitkul T, Wittayakun J, Ruksakulpiwat C, Ruksakulpiwat Y (2022) Nano-hydroxyapatite from white seabass scales as a bio-filler in polylactic acid biocomposite: preparation and characterization. Polymers 14:4158. 10.3390/polym14194158 36236110 10.3390/polym14194158 PMC9571318

[CR16] Kraakman NJ, Rocha-Rios J, Van Loosdrecht MC (2011) Review of mass transfer aspects for biological gas treatment. Appl Microbiol Biot 91:873–886. 10.1007/s00253-011-3365-5 10.1007/s00253-011-3365-5 PMC3145080 21701986

[CR17] Łebkowska M, Narożniak-Rutkowska A, Pajor E (2013) Effect of a static magnetic field of 7mT on formaldehyde biodegradation in industrial wastewater from urea–formaldehyde resin production by activated sludge. Bioresource Technol 132:78–83. 10.1016/j.biortech.2013.01.020 10.1016/j.biortech.2013.01.020 23395758

[CR18] Łebkowska M, Rutkowska-Narożniak A, Pajor E, Tabernacka A, Załęska-Radziwiłł M (2018) Impact of a static magnetic field on biodegradation of wastewater compounds and bacteria recombination. Environ Sci Pollut R 25:22571–22583. 10.1007/s11356-018-1943-0 10.1007/s11356-018-1943-0 29845547

[CR19] Li J, Yi Y, Cheng X, Zhang D, Irfan M (2015) Study on the effect of magnetic field treatment of newly isolated *Paenibacillus* sp. Bot Stud 56:1–9. 10.1186/s40529-015-0083-9 28510811 10.1186/s40529-015-0083-9 PMC5430345

[CR20] Liao BQ, Allen DG, Droppo IG, Leppard GG, Liss SN (2001) Surface properties of sludge and their role in bioflocculation and settleability. Water Res 35:339–350. 10.1016/S0043-1354(00)00277-3 11228985 10.1016/s0043-1354(00)00277-3

[CR21] Liu Y, Liu Q, Li J, Ngo HH, Guo W, Hu J, Gao M, Wang Q, Hou Y (2018) Effect of magnetic powder on membrane fouling mitigation and microbial community/composition in membrane bioreactors (MBRs) for municipal wastewater treatment. Bioresource Technol 249:377–385. 10.1016/j.biortech.2017.10.027 10.1016/j.biortech.2017.10.027 29055214

[CR22] Lu Y, Wang X, Liu W, Li E, Cheng F, Miller JD (2019) Dispersion behavior and attachment of high internal phase water-in-oil emulsion droplets during fine coal flotation. Fuel 253:273–282. 10.1016/j.fuel.2019.05.012

[CR23] Ma X, Duan D, Wang X, Cao J, Qiu J, Xie B (2021) Degradation of *Rhodococcus**erythropolis* SY095 modified with functional magnetic Fe_3_O_4_ nanoparticles. P Roy Soc B-Biol Sci 8:211172. 10.1098/rsos.211172 10.1098/rsos.211172 PMC8692970 34950489

[CR24] Mahto KU, Vandana, Priyadarshanee M, Samantaray DP, Das S (2022) Bacterial biofilm and extracellular polymeric substances in the treatment of environmental pollutants: beyond the protective role in survivability. J Clean Prod 379:134759. 10.1016/j.jclepro.2022.134759

[CR25] Mohammadi-Jam S, Waters KE, Greenwood RW (2022) A review of zeta potential measurements using electroacoustics. Adv Colloid Interface Sci 309:102778. 10.1016/j.cis.2022.102778 36209685 10.1016/j.cis.2022.102778

[CR26] Moore RL (1979) Biological effects of magnetic fields: studies with microorganisms. Can J Microbiol 25:1145–1151. 10.1139/m79-178 119572 10.1139/m79-178

[CR27] Nadell CD, Drescher K, Wingreen NS, Bassler BL (2015) Extracellular matrix structure governs invasion resistance in bacterial biofilms. ISME J 9:1700–1709. 10.1038/ismej.2014.246 25603396 10.1038/ismej.2014.246 PMC4511925

[CR28] Okano H (2008) Effects of static magnetic fields in biology: role of free radicals. Front BiosciLandmrk 13:6106–6125. 10.2741/3141 10.2741/3141 18508647

[CR29] Oliveira LCA, Rios RVRA, Fabris JD, Garg V, Sapag K, Lago RM (2002) Activated carbon/iron oxide magnetic composites for the adsorption of contaminants in water. Carbon 40:2177–2183. 10.1016/S0008-6223(02)00076-3

[CR30] Quan Y, Wu H, Yin Z, Fang Y, Yin C (2017) Effect of static magnetic field on trichloroethylene removal in a biotrickling filter. Bioresour Technol 239:7–16. 10.1016/j.biortech.2017.04.121 28500890 10.1016/j.biortech.2017.04.121

[CR31] Quan Y, Wu H, Guo C, Han Y, Yin C (2018) Enhancement of TCE removal by a static magnetic field in a fungal biotrickling filter. Bioresource Technol 259:365–372. 10.1016/j.biortech.2018.03.031 10.1016/j.biortech.2018.03.031 29574317

[CR32] Rakoczy R, Lechowska J, Kordas M, Konopacki M, Fijałkowski K, Drozd R (2017) Effects of a rotating magnetic field on gas-liquid mass transfer coefficient. Chem Eng J 327:608–617. 10.1016/j.cej.2017.06.132

[CR33] Ramirez M, Gómez JM, Aroca G, Cantero D (2009) Removal of hydrogen sulfide and ammonia from gas mixtures by co-immobilized cells using a new configuration of two biotrickling filters. Water Sci Technol 59:1353–1359. 10.2166/wst.2009.105 19381001 10.2166/wst.2009.105

[CR34] Ren ZJ, Wang PF, Tian JY, Zhang ZL (2019) Effects of a low-strength magnetic field on the characteristics of activated sludge for membrane fouling mitigation. RSC Adv 9:9180–9186. 10.1039/c8ra10013f 35517664 10.1039/c8ra10013f PMC9061976

[CR35] Ren X, Qu R, Liu S, Zhao H, Wu W, Song H, Zheng C, Wu X, Gao X (2020) Synthesis of zeolites from coal fly ash for the removal of harmful gaseous pollutants: a review. Aerosol Air Qual Res 20:1127–1144. 10.4209/aaqr.2019.12.0651

[CR36] Rosenberg M, Kjelleberg S (1986) Hydrophobic interactions: role in bacterial adhesion. In: Marshall KC (ed) Advances in Microbial Ecology, Advances in Microbial Ecology. Springer, pp 353–393. 10.1007/978-1-4757-0611-68

[CR37] Rybarczyk P, Szulczyński B, Gębicki J, Hupka J (2019) Treatment of malodorous air in biotrickling filters: a review. Biochem Eng J 141:146–162. 10.1016/j.bej.2018.10.014

[CR38] San-Valero P, Gabaldón C, Penya-roja JM, Quijano G (2017) Enhanced styrene removal in a two-phase partitioning bioreactor operated as a biotrickling filter: towards full-scale applications. Chem Eng J 309:588–595. 10.1016/j.cej.2016.10.054

[CR39] Saravanan P, Pakshirajan K, Saha P (2008) Growth kinetics of an indigenous mixed microbial consortium during phenol degradation in a batch reactor. Bioresource Technol 99:205–209. 10.1016/j.biortech.2006.11.045 10.1016/j.biortech.2006.11.045 17236761

[CR40] Sennour R, Mimane G, Benghalem A, Taleb S (2009) Removal of the persistent pollutant chlorobenzene by adsorption onto activated montmorillonite. Appl Clay Sci 43:503–506. 10.1016/j.clay.2008.06.019

[CR41] Shi J, Liang Z, Dai X (2023) Enhanced biological phosphorus and nitrogen removal by high-concentration powder carriers: extracellular polymeric substance, microbial communities, and metabolic pathways. Environ Sci Pollut R 30:4010–4022. 10.1007/s11356-022-22363-8 10.1007/s11356-022-22363-8 35963965

[CR42] Tan L, Shao Y, Mu G, Ning S, Shi S (2020) Enhanced azo dye biodegradation performance and halotolerance of Candida tropicalis SYF-1 by static magnetic field (SMF). Bioresource Technol 295:122283. 10.1016/j.biortech.2019.122283 10.1016/j.biortech.2019.122283 31669874

[CR43] Vandana, Priyadarshanee M, Das S (2023) Bacterial extracellular polymeric substances: biosynthesis and interaction with environmental pollutants. Chemosphere 332:138876. 10.1016/j.chemosphere.2023.138876 37164199 10.1016/j.chemosphere.2023.138876

[CR44] Wang C, Xi J-Y, Hu H-Y, Yao Y (2009) Advantages of combined UV photodegradation and biofiltration processes to treat gaseous chlorobenzene. J Hazard Mater 171:1120–1125. 10.1016/j.jhazmat.2009.06.129 19616379 10.1016/j.jhazmat.2009.06.129

[CR45] Wang Y, Wei H, Li Z (2018) Effect of magnetic field on the physical properties of water. Results Phys 8:262–267. 10.1016/j.rinp.2017.12.022

[CR46] Wang M, Li J, Ning S, Fu X, Wang X, Tan L (2022a) Simultaneously enhanced treatment efficiency of simulated hypersaline azo dye wastewater and membrane antifouling by a novel static magnetic field membrane bioreactor (SMFMBR). Sci Total Environ 821:153452. 10.1016/j.scitotenv.2022.153452 35093373 10.1016/j.scitotenv.2022.153452

[CR47] Wang R, Wei B, Zhu J, Zhao X, Yu G (2022b) Study on the hydrodynamic performance and treatment effect of a modified biological carrier in wastewater treatment. Sci Total Environ 844:156974. 10.1016/j.scitotenv.2022.156974 35764159 10.1016/j.scitotenv.2022.156974

[CR48] Wang Q, Lin Y, Peng L, Wang Y, Ma S, Ren H, Xu K (2023) Weak magnetic field enhances waste molasses-driven denitrification during wastewater treatment. Bioresour Technol 387:129697. 10.1016/j.biortech.2023.129697 37598801 10.1016/j.biortech.2023.129697

[CR49] Wu X, Lin Y, Wang Y, Wu S, Li X, Yang C (2022) Enhanced removal of hydrophobic short-chain n-alkanes from gas streams in biotrickling filters in presence of surfactant. Environ Sci Technol 56:10349–10360. 10.1021/acs.est.2c02022 35749664 10.1021/acs.est.2c02022

[CR50] Wu X, Lin Y, Wang Y, Wu S, Yang C (2023) Volatile organic compound removal via biofiltration: influences, challenges, and strategies. Chem Eng J 471:144420. 10.1016/j.cej.2023.144420

[CR51] Yang Y, Qiu X, Sun Y, Wang Y, Wang J, Li Y, Liu C (2018) Development of bioabsorbable polylactide membrane with controllable hydrophilicity for adjustment of cell behaviours. R Soc Open Sci 5:170868. 10.1098/rsos.170868 29410803 10.1098/rsos.170868 PMC5792880

[CR52] Yavuz H, Çelebi SS (2000) Effects of magnetic field on activity of activated sludge in wastewater treatment. Enzyme Microb Tech 26:22–27. 10.1016/S0141-0229(99)00121-0

[CR53] You J, Yu J, Zhang S, Chen J, Chen D (2023) Performance and mechanism of innovative two-phase partitioning microbial fuel cell for effective propanethiol treatment. Chem Eng J 453:139731. 10.1016/j.cej.2022.139731

[CR54] Yuan D, Wang Y (2013) Effects of solution conditions on the physicochemical properties of stratification components of extracellular polymeric substances in anaerobic digested sludge. J Environ Sci- China 25:155–162. 10.1016/S1001-0742(12)60038-2 23586310 10.1016/s1001-0742(12)60038-2

[CR55] Zhang C, Jia L, Wang S, Qu J, Li K, Xu L, Shi Y, Yan Y (2010) Biodegradation of beta-cypermethrin by two Serratia spp. with different cell surface hydrophobicity. Bioresource Technol 101:3423–3429. 10.1016/j.biortech.2009.12.083 10.1016/j.biortech.2009.12.083 20116237

[CR56] Zhang LL, Leng SQ, Zhu RY, Chen JM (2011) Degradation of chlorobenzene by strain Ralstonia pickettii L2 isolated from a biotrickling filter treating a chlorobenzene-contaminated gas stream. Res Chem Intermediat 91:407–415. 10.1007/s00253-011-3255-x 10.1007/s00253-011-3255-x 21499764

[CR57] Zhang H, Yu H, Zhang L, Song L (2015) Stratification structure of polysaccharides and proteins in activated sludge with different aeration in membrane bioreactor. Bioresource Technol 192:361–366. 10.1016/j.biortech.2015.05.025 10.1016/j.biortech.2015.05.025 26056777

[CR58] Zhang K, Sun P, Faye MCAS, Zhang Y (2018) Characterization of biochar derived from rice husks and its potential in chlorobenzene degradation. Carbon 130:730–740. 10.1016/j.carbon.2018.01.036

[CR59] Zhou L, Li G, An T, Li Y (2010) Synthesis and characterization of novel magnetic Fe_3_O_4_/polyurethane foam composite applied to the carrier of immobilized microorganisms for wastewater treatment. Res Chem Intermediat 36:277–288. 10.1007/s11164-010-0134-5

[CR60] Zieliński M, Rusanowska P, Dębowski M, Hajduk A (2018) Influence of static magnetic field on sludge properties. Sci Total Environ 625:738–742. 10.1016/j.scitotenv.2017.12.226 29306162 10.1016/j.scitotenv.2017.12.226

[CR61] Zikmanis P, Shakirova L, Auzina L, Andersone I (2007) Hydrophobicity of bacteria Zymomonas mobilis under varied environmental conditions. Process Biochem 42:745–750. 10.1016/j.procbio.2007.01.00

