# Supplementary material for: Enhanced chlorobenzene removal by internal magnetic field through initial cell adhesion and biofilm formation
Source: Appl Microbiol Biotechnol. 2024 Jan 22;108:159. doi: 10.1007/s00253-024-13001-z (PMC10803521; doi:10.1007/s00253-024-13001-z)
Supplement: Supplementary file 1 [file 253_2024_13001_MOESM1_ESM.pdf]

Applied Microbiology and Biotechnology

Enhanced chlorobenzene removal by internal magnetic field through initial cell adhesion and biofilm formation

Dong-zhi Chen[a,b,d], Jinfeng Qiu[a,b,c], Haimin Sun[e], Yanting Liu[f], Jiexu Ye[a], Jian-Meng Chen[a], Lichao Lu[b,d,*].

[a] *College of Environment, Zhejiang University of Technology, Hangzhou 310032, China*

[b] *School of Petrochemical Engineering and Environment, Zhejiang Ocean University, Zhoushan 316004, China*

[c] *Collaborative Innovation Center of Yangtze River Delta Region Green Pharmaceuticals, Zhejiang University of Technology, Hangzhou, 310032, China*

[d] *Zhejiang Provincial Key Laboratory of Petrochemical Pollution Control, Zhoushan 316004, China*

[e] *Zhejiang Zhonglan Environmental Technology Co., Ltd., Wenzhou 325000, China*

[f] *Yali High School, No. 428 Laodong Western Road, Changsha, Hunan, P.R. China, 410007*

*Corresponding author:

Lichao Lu

Tel: 86-0580-2552170

E-mail: lu_lc21@zjou.edu.cn

Supplementary materials

1. Performance evaluation of chlorobenzene

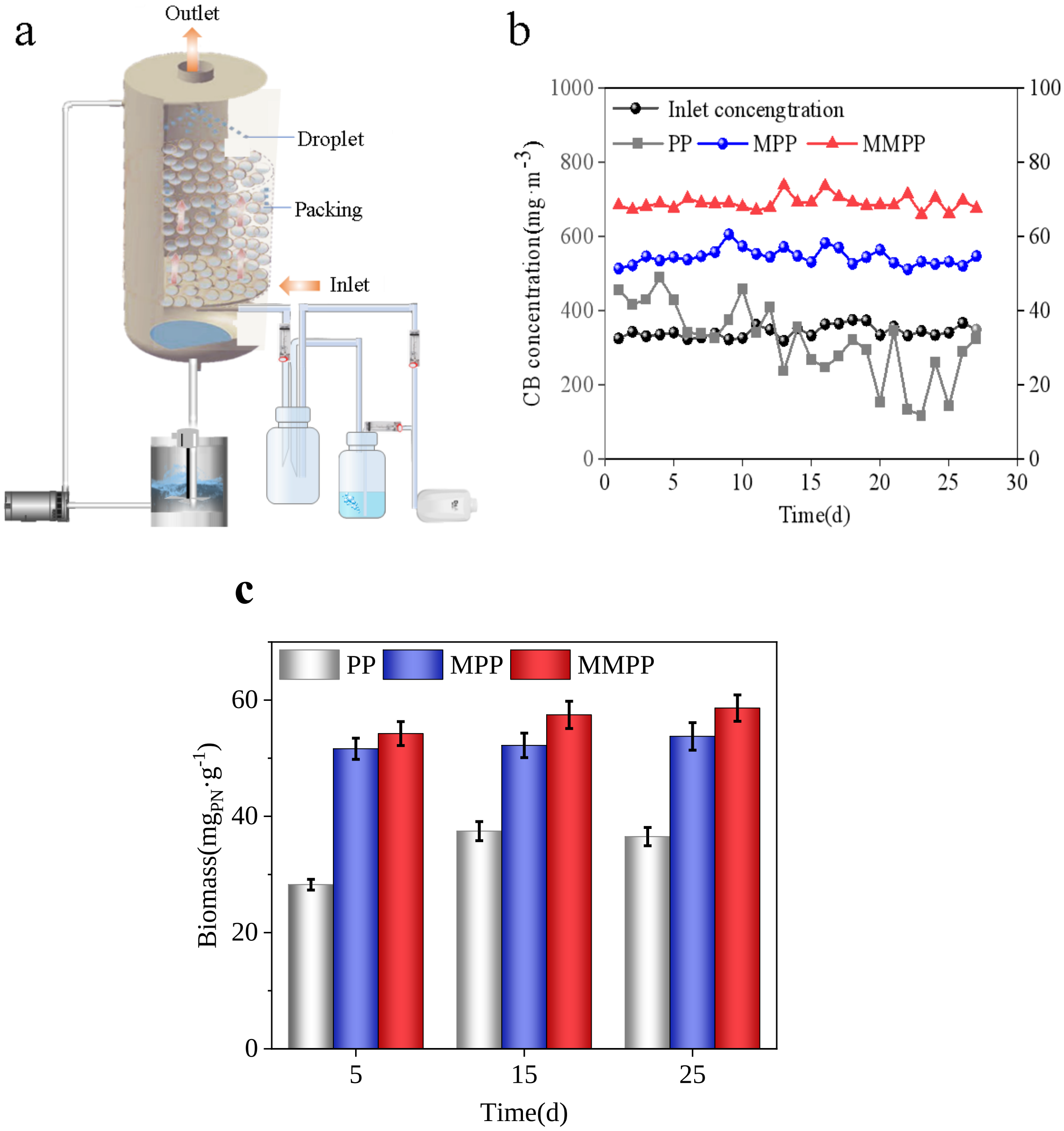

Fig. S1 Craft of the laboratory-scale bio-trickling filter (BTF) system: (a) the BTF Schematic, (b) the removal efficiency (RE) of CB with inlet concentration of 300-400 $mg.m^{-3}$ at EBRT of 60 s in BTF1 (PP), BTF2 (MPP) and BTF3 (MMPP). The bio-trickling filter was constructed as a column by steel with the inside diameter of 150 mm and the height of 570 mm. The inoculum was the CB-degrading bacterium, *Ralstonia* sp. XZW-1. The liquid mineral medium was used as the nutrient with a spray rate of

5.4 L/h. The data was recorded after 14 days starting-up period. (c) the biomass (presented as protein) on the packing surface in the BTF.

2.Excitation–emission matrix spectra

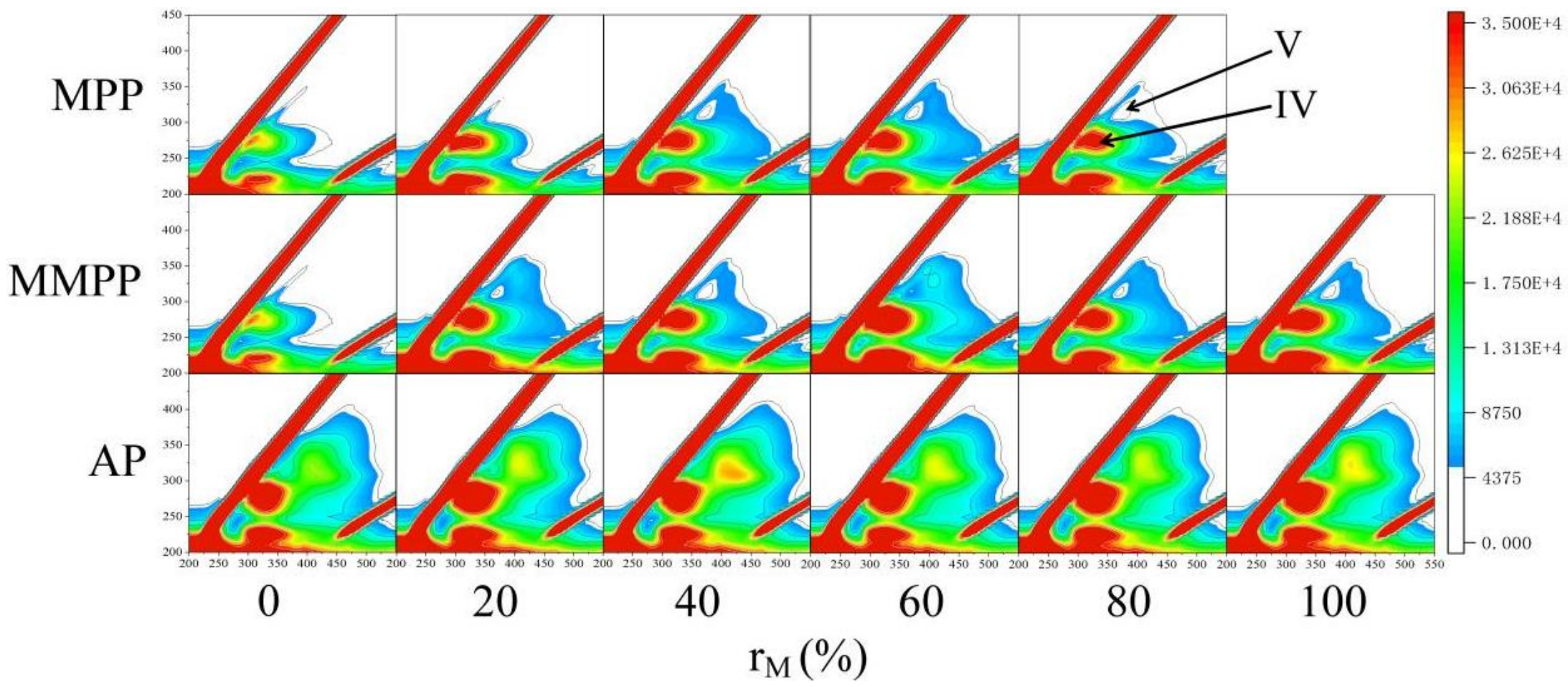

Fig. S2 Cellular characteristics under the different MFI： the three-dimensional excitation–emission matrix fluorescence spectra, where the proteoid substances (IV) represent the microbial metabolism and the humic acid (V) represent the microbial apoptosis.

3.Biodegradation kineic analysis of CB

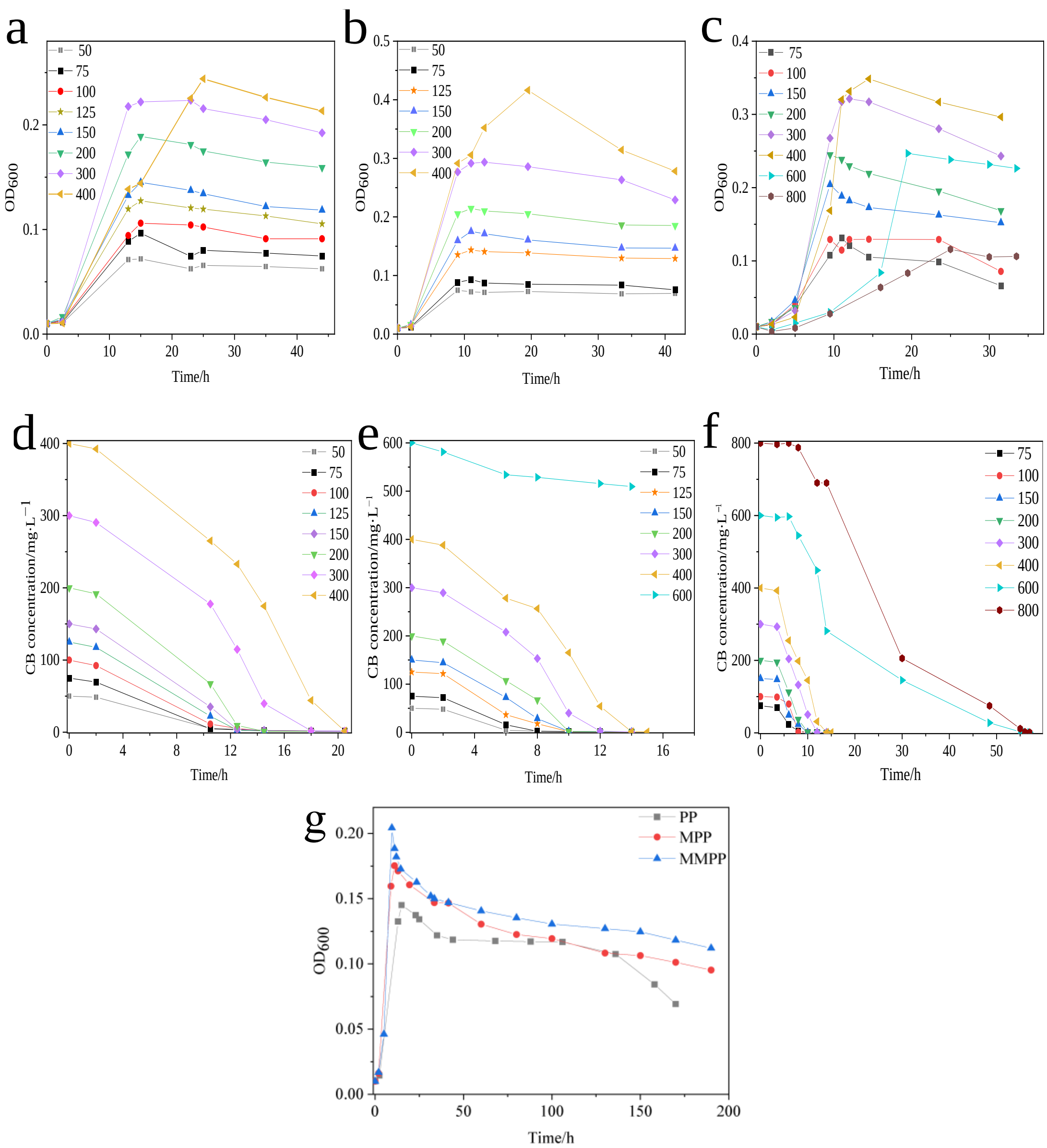

Fig. S3 The experimental data of the microbial growth and CB degradation for kinetic analysis: (a) Microbial growth cure for the PP, (b) Microbial growth cure for the MPP, (c) Microbial growth cure for the MMPP, (d) microbial biodegradation cure of the PP, (e) microbial biodegradation cure of the MPP, (f) microbial biodegradation cure of the

MMPP, (g) microbial decay cure for the packings with the concentration of 150 mg·L^{-1}.

Table S1 The calculated kinetic parameters

Packings	μ_{max} (h^{-1})	V_{max} (h^{-1})	K_S (mg·L^{-1})	K_I (mg·L^{-1})	K_d (h^{-1})
PP	0.197	0.279	182.262	284.663	0.0036
MPP	0.276	0.350	107.350	157.855	0.0033
MMPP	0.470	0.455	100.003	115.249	0.0027
